# Immortalization of Adipose‐Derived Stromal Cells From Japanese Black Cattle

**DOI:** 10.1002/cbin.70194

**Published:** 2026-08-06

**Authors:** Rei Sasaki, Hanami Matsukura, Ryo Meguro, Lanlan Bai, Ken Sawai, Eriko Sugano, Hiroshi Tomita, Tohru Kiyono, Tomokazu Fukuda

**Affiliations:** ^1^ Division of Agriculture Iwate University Morioka Japan; ^2^ Division of Collaborative Research and Development, Exploratory Oncology Research and Clinical Trial Center National Cancer Center Chiba Japan; ^3^ Department of Cancer Cell Research Sasaki Institute, Sasaki Foundation Tokyo Japan

**Keywords:** adipocyte differentiation, Adipose‐Derived Stromal Cells, cell cycle regulator, immortalization, Japanese Black cattle, SV40 large T antigen

## Abstract

Adipose tissue plays a critical role in determining the characteristic palatability of Japanese Black cattle beef, including its distinctive aroma, tenderness, and flavor. These quality traits are closely associated with adipogenic differentiation and lipid metabolism; however, the underlying molecular mechanisms remain poorly understood. Adipose‐derived stromal cells (ASCs) residing within adipose tissue possess the capacity to differentiate into adipocytes and other mesenchymal lineages and are, therefore, considered valuable experimental resources for adipose tissue research. Nevertheless, primary cells are subject to several limitations, including a restricted proliferative lifespan caused by culture‐induced stress and reduced experimental reproducibility due to inter‐individual variability, which hamper their use in long‐term *in vitro* analyses. In this study, we immortalized ASCs derived from Japanese Black cattle using the K4DT method, which involves the introduction of the R24C mutant form of cyclin‐dependent kinase 4 (CDK4^R24C^), Cyclin D1, and telomerase reverse transcriptase. The established bovine ASCs (bASCs) [bASCs‐K4DT] exhibited a stable proliferative capacity during long‐term culture while retaining their ability to undergo adipogenic differentiation. These immortalized bASCs may serve as a useful *in vitro* model for elucidating the molecular mechanisms underlying adipocyte differentiation and formation of breed‐specific aroma and flavor characteristics.

**Abbreviations**
bASC: bovine adipose‐derived stromal cell
CDK4: cyclin‐dependent kinase 4
C/EBP: CCAAT/enhancer‐binding protein
EGFP: enhanced green fluorescent protein
HPV: human papillomavirus
HRP: horseradish peroxidase
hTERT: human derived telomerase reverse transcriptase
IBMX: 3‐isobutyl‐1‐methylxanthine
KCl: potassium chloride
MUFA: monounsaturated fatty acid
PD: population doubling
PPARγ: peroxisome proliferator‐activated receptor gamma
PTFE: polytetrafluoroethylene
SCD: stearoyl‐CoA desaturase
SV40T: simian virus 40 large T antigen
SVF: stromal vascular fraction
TERT: telomerase reverse transcriptase
TGF‐β: transforming growth factor‐β
TSC1: tuberous sclerosis complex 1

## 1 Introduction

In recent years, Japanese Black cattle have become highly valued both domestically and internationally because of their superior beef palatability. One of the defining characteristics of this breed is its markedly higher degree of marbling compared with other cattle breeds, which contributes to enhanced its umami flavor and sweetness, as well as the formation of the characteristic “Wagyu aroma” (Gotoh et al. 2014; Platter et al. 2005). These traits are closely associated with a high proportion of unsaturated fatty acids, particularly oleic acid, in intramuscular fat (Gotoh et al. 2014). Previous studies have suggested that formation of marbling is regulated by a complex molecular network and signaling pathways, including transforming growth factor‐β (TGF‐β) signaling and Ras/Rho family pathways (Ueda et al. 2021). However, detailed molecular mechanisms underlying these processes remain unclear.

Adipose‐derived stromal cells (ASCs) are undifferentiated mesenchymal cells isolated from adipose tissue and possess the capacity to differentiate into adipocytes (Bunnell 2021). Owing to this property, ASCs have been widely used as a useful *in vitro* research model that exhibits an adipogenic differentiation capacity for analyses of lipolysis and fatty acid metabolism (Jurek et al. 2020; Yue et al. 2018). However, primary ASCs are subject to several limitations such as a restricted proliferative lifespan due to cellular senescence and low reproducibility caused by inter‐individual variability (Hayflick and Moorhead 1961; Zhu et al. 2024). These constraints limit their applicability in high‐precision analyses, such as comprehensive gene expression profiling and metabolic pathway studies.

To date, one of the approaches used for cellular immortalization involves the introduction of the simian virus 40 (SV40) large T antigen. However, this method is known to be associated with a progressive decline in cellular differentiation capacity with increasing passage numbers, which is accompanied by reduced expression of adipogenic genes such as peroxisome proliferator‐activated receptor gamma (PPARγ) and C/EBPα (Church et al. 2015). Therefore, the establishment of stable cell lines capable of long‐term proliferation while retaining their adipogenic potential is essential to elucidate the mechanisms underlying adipocyte differentiation and aroma‐related lipid metabolism in Japanese Black cattle.

We have previously reported that the K4DT method, which involves the introduction of a mutant form of CDK4 (CDK4^R24C^) resistant to inhibition by p16^INK4a^, Cyclin D1, and telomerase reverse transcriptase (TERT), enables the immortalization of various primary cultured cells (Bai et al. 2025; Kikuchi et al. 2023). However, whether bovine adipose‐derived stromal cells (ASCs) can be immortalized using the K4DT method while retaining their adipogenic differentiation potential has not been investigated. Compared with primary ASCs, immortalized ASCs provide several advantages, including stable proliferative capacity, improved experimental reproducibility, and suitability for long‐term culture and repeated genetic or pharmacological manipulation. These characteristics suggest that immortalized ASCs may provide a valuable experimental platform for investigating the molecular mechanisms regulating adipogenic differentiation and lipid metabolism. These characteristics suggest that immortalized ASCs may provide a valuable experimental platform for investigating the molecular mechanisms regulating adipogenic differentiation and lipid metabolism. Such studies are particularly relevant to Japanese Black cattle, in which adipogenesis is closely associated with intramuscular fat deposition, a major determinant of beef quality. Therefore, establishing immortalized ASCs from Japanese Black cattle may provide a stable and reproducible in vitro model for investigating the regulatory mechanisms underlying adipogenesis and lipid accumulation in this economically important breed. In this study, we applied the K4DT method to primary ASCs isolated from Japanese Black cattle and established immortalized cell lines with particular emphasis on their adipogenic differentiation potential.

## 2 Materials and Methods

### 2.1 Primary Cell Culture

ASCs were isolated from the parametrium adipose tissue of female two Japanese Black cattle at a commercial slaughterhouse. In brief, Adipose tissue was minced into small fragments and digested with collagenase. After enzymatic digestion, the cell suspension was filtered and centrifuged to obtain the stromal vascular fraction (SVF). The resulting cell pellet was resuspended in culture medium and seeded into culture dishes. After removal of non‐adherent cells by medium replacement, the adherent fibroblast‐like cells were expanded and used as adipose‐derived stromal cells (ASCs) (Si et al. 2019).

### 2.2 Lentiviral and Retroviral Vector Production

Lentiviral vectors expressing human derived CDK4^R24C^, Cyclin D1, telomerase reverse transcriptase (hTERT), and enhanced green fluorescent protein (EGFP) were generated by co‐transfecting 293 T cells. The origin of exogenous genes were all derived from human. In brief, the plasmids CSII‐CMV‐CDK4^R24C^, CSII‐CMV‐Cyclin D1, CSII‐CMV‐TERT, or CSII‐CMV‐EGFP, together with the packaging plasmids, pCMV‐VSVG‐RSV‐Rev and pCAG‐HIV‐gp (backbone and packaging plasmids were provided by RIKEN BioResource Center using the lipofection method). Retroviral vectors expressing the simian virus 40 (SV40) large T antigen and EGFP were generated by co‐transfecting 293 T cells with QCXIN‐SV40T or QCXIN‐EGFP along with packaging plasmids pCL‐gag‐pol and pCMV‐VSVG‐RSV‐Rev. Culture supernatants containing each recombinant virus were collected 48 h post‐transfection and filtered through a 0.45‐μm filter. The viral supernatants were then mixed with a PEG6000‐based concentrating solution at a ratio of 3:1 (supernatant: concentrating solution) and centrifuged at 3,500 rpm for 1 h to concentrate the viruses. The resulting viral pellets were resuspended in 1 mL of culture medium and used for infection.

### 2.3 Cell Immortalization

The bovine adipose‐derived stromal cells (bASCs) were cultured in Dulbecco's Modified Eagle Medium (DMEM, high glucose) supplemented with 10% fetal bovine serum (FBS) and antibiotics. The antibiotics included ciprofloxacin (final concentration, 10 μg/mL), amphotericin B (0.25 μg/mL), and penicillin–streptomycin (10 U/mL and 10 μg/mL, respectively). The bASCs were seeded at 1 × 10^5^ cells per well in 6‐well plates and, after 24 h of incubation, the primary cells were infected with concentrated recombinant viruses in the presence of 8 μg/mL of polybrene. Cells transduced with viruses expressing CDK4^R24C^, Cyclin D1, and TERT; those expressing CDK4^R24C^ and Cyclin D1; and those expressing SV40T were named K4D cells, K4DT cells, and SV40 cells, respectively, consistent with the introduced genes.

### 2.4 PCR

Genomic DNA was extracted from parental and transduced cells using the NucleoSpin Tissue kit (Takara Bio, Shiga, Japan) according to the manufacturer's instructions. Each genomic DNA was subjected to genomic PCR using the KOD FX Neo PCR enzyme (TOYOBO, Osaka, Japan). We chose to use the tuberous sclerosis Tuberous sclerosis complex 1 (TSC1) as a positive control, since the TSC1 gene is highly conserved across mammalian genomes and is present at a unique locus. The primers used were as follows: CDK4R24C, TF1066 (5´‐CTTCCCATCAGCACAGTTCGTGAGG‐3´) and TF1067(5´‐AAAGATTTTGCCCAACTGGTCGGCTTC‐3´); CycinD1, TF1064 (5´‐CCCGATGCCAACCTCCTCAACGAC‐3´) and TF1065 (5´‐ATGATCTGTTTGTTCTCCTCCGCCTCTG‐3´); TERT, TF961(5´‐CTGCTCCTGCGTTTGGTGGATGATT‐3´) and TF962 (5´‐GTCC TGAGTGACCCCAGGAGTGGCA‐3´); SV40, TF1068 (5´‐ATGTATAGTGCCTTGACTAGAGATCCAA‐3´) and TF1069 (5´‐CCAGCCATCCATTCTTCTATGTC‐3´); and TSC1, ll43 (5´‐AGTCATTCCATGCCGAGGAG‐3´) and ll44 (5´‐CAGGATCCCTGACTGCGAC‐3´).

### 2.5 Western Blot Analysis

For western blotting, total protein was obtained following a previously published procedure (Fukuda et al. 2008). Primary antibodies used were mouse anti‐CDK4 (1:2000 dilution, sc‐56277, MBL, Tokyo, Japan), anti‐Cyclin D1 monoclonal (1:5000 dilution, #553, MBL), and anti‐α‐tubulin (1:1000 dilution, sc‐32293, Santa Cruz, TX, USA). S econdary antibodies used were anti‐mouse IgG with horseradish peroxidase (HRP) (1:3000, code 330, MBL, Tokyo, Japan) and anti‐rabbit IgG with horseradish peroxidase (HRP) (1:5000, code 458, MBL, Tokyo, Japan). Immunoreactive bands were visualized using the Pierce chemiluminescent substrate (Prod. No. NCI3109; Thermo Scientific, MA, USA), and images were captured using the LAS‐4000 Mini System (Fujifilm, Tokyo, Japan).

### 2.6 Cell Population Doubling

Parental and transduced cells were seeded at a density of 5 × 10^4^ cells/well in a 6‐well plate (initial number of cells, N_0_). When one type of the cultured cells reached confluence, all cell types were harvested, counted (final number of cells, N_f_), and serially passaged. Population doubling (PD) was calculated as the cumulative sum of log_2_^*N*f/*N*0^. The population doubling data were obtained from three independent experiments (*n* = 3).

### 2.7 Cell Cycle Analysis

Cell cycle analysis was performed at passage 12 or 13 using the Muse Cell Analyzer (Millipore, Burlington, MA, USA) according to the manufacturer's instructions. The principle of the kit is staining with propidium iodide along with RNase treatment. Each experimental group consisted of six replicates. Statistical significance among groups was evaluated using the nonparametric Steel–Dwass test.

### 2.8 Karyotype Analysis

To examine karyotypes of the cells, they were cultured in the presence of 10 μg/mL of Colcemid solution overnight to promote cell metaphase. Next, the cells were harvested, treated with 75 mM KCl as a hypotonic solution, and fixed using a mixture of acetic acid and methanol. The treated cell samples were spread on glass slides and stained with Giemsa solution. The numbers of chromosomes present in over 70 mitotic cells w visually scored.

### 2.9 Induction of Differentiation in bASCs

To induce adipogenic differentiation, cells were seeded at a density of 5 × 10^4^ cells/well and cultured for 2 days until they reached 70%–90% confluence, after which the medium was replaced with an adipogenic differentiation medium [containing high‐glucose DMEM supplemented with 10 μg/mL insulin (FUJIFILM Wako), 1 μM dexamethasone (FUJIFILM Wako), 0.5 mM IBMX (FUJIFILM Wako), 5 μM rosiglitazone (Funakoshi), and 3% FBS]. Four cell types were subjected to differentiation: two parental bASCs (bASC1 and bASC2, both at passage 2), K4DT cells (at passages 17 and 19) and SV40 cells (both at passage 17). Three days after induction, the medium was replaced with a maintenance medium [containing high‐glucose DMEM supplemented with 10 μg/mL insulin and 3% FBS], followed by re‐exposure to the adipogenic differentiation medium on day 4. The cells were then cultured for 8 days to evaluate adipogenic differentiation.

Following differentiation, intracellular lipid accumulation was assessed using Oil Red O staining. Cells were fixed with 4% paraformaldehyde for 20 min at room temperature (25°C) and washed twice with PBS. A 0.3% Oil Red O stock solution was prepared by dissolving Oil Red O powder (FUJIFILM Wako) in 2‐propanol. Before use, the stock solution was diluted with distilled water (3:2) and filtered through a 0.22‐μM PTFE membrane to prepare a working solution. Fixed cells were incubated with the working solution for 15 min at room temperature. After staining, the cells were washed with PBS and imaged using a microscope (KEYENCE).

### 2.10 Image Analysis

Oil Red O‐stained cells were examined under a microscope (KEYENCE), and images were captured at × 10 magnification. Image analysis was performed using ImageJ software (National Institutes of Health, Bethesda, MD, USA). The stained images were binarized with threshold function of software. The same value of threshold has applied into the control and differentiated cells. The amount of positive area was detected with measure function of the software. 15 images from triplicated samples were processed into the quantitative data. Data are presented as the mean ± SE of three independent experiments.

## 3 Results

### 3.1 Immortalization of Bovine Adipose‐Derived Stem Cells (bASCs)

**Figure 1:**
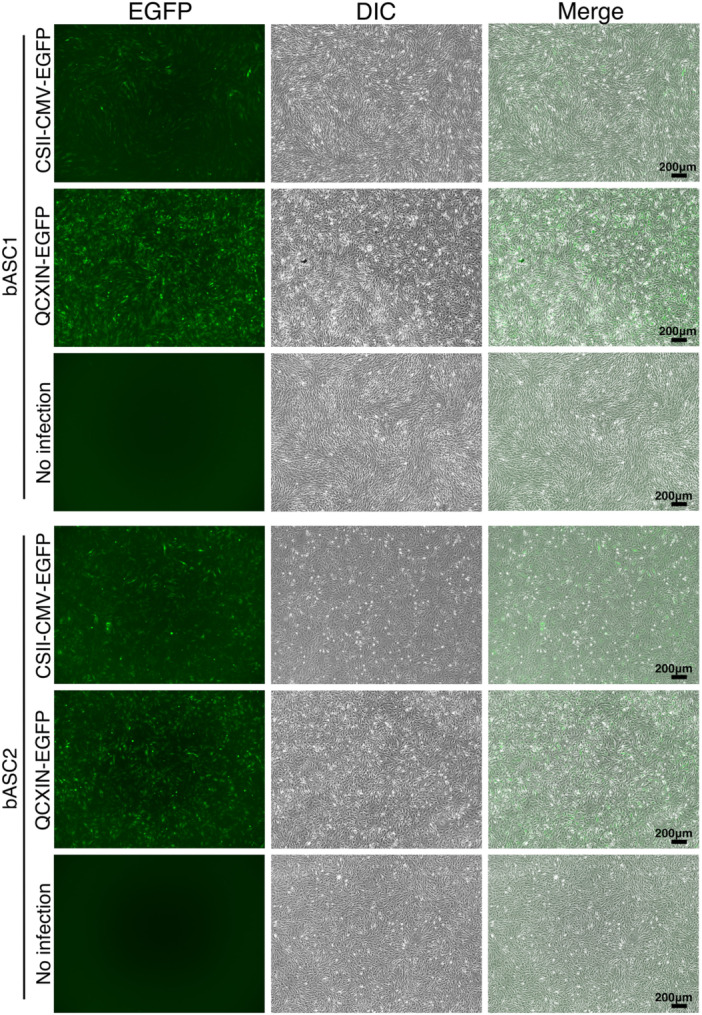
Evaluation of viral transduction efficiency in bovine‐derived cells (bASC1 and bASC2). Representative fluorescence images of cells transduced with recombinant viral vectors expressing EGFP are shown. All the fluorescence images were acquired using identical exposure settings. Scale bar = 200 μM. CSII‐CMV‐EGFP (upper panel), QCXIN‐EGFP (middle panel), and non‐transduced control (bottom panel).

To estimate transduction efficiency of the lentiviral vectors (CDK4^R24C^, and Cyclin D1, TERT) and the retroviral vector (SV40 large T antigen), recombinant viruses expressing EGFP were used to assess efficiency of infection in adipose‐derived stromal cells from two cattle (designated bASC1 and bASC2) as the surrogate marker. At 96 h post‐infection, a high number of EGFP‐positive cells was observed under a fluorescence microscope (Figure 1). These findings suggest that CDK4^R24C^, Cyclin D1, TERT, and SV40 were efficiently introduced into bASCs.

### 3.2 Detection of the Expression of the Introduced Genes

**Figure 2:**
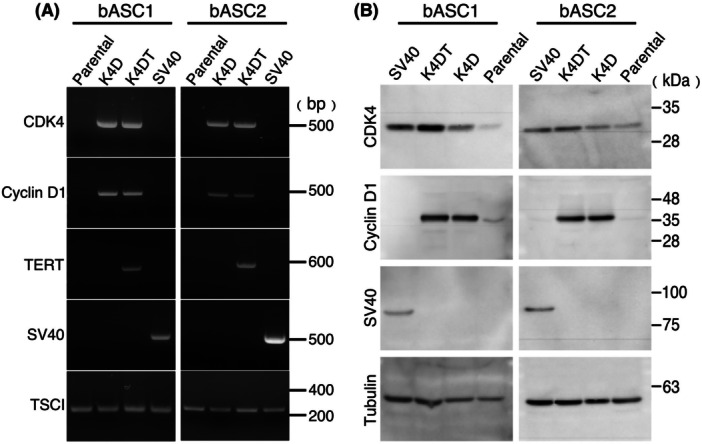
Detection of exogenous gene expression in bovine‐derived cells (bASC1 and bASC2). (A) Presence of the introduced genes in parental, K4D, K4DT and SV40 cells was examined by PCR. Tuberous sclerosis complex 1 (TSC1) as used as an internal control. (B) Protein expression of the introduced genes was confirmed by western blotting. Tubulin was used as a loading control.

To verify the integration of the introduced genes into the genome of the target cells and their expression, PCR and western blot analysis were performed. Amplification products of the expected sizes for CDK4^R24C^, Cyclin D1, TERT, and SV40 were detected using PCR analysis (Figure 2A and Figure S1A, S1B). Furthermore, western blot analysis revealed clear protein bands corresponding to each gene product, indicating that the introduced genes were also expressed at the protein level (Figure 2B and Figure S2A, S2B). Taken together, these results indicate that introduction of the genes was successfully achieved in bASCs, and that six cell lines corresponding to “K4D”, “K4DT”, and “SV40” cells were successfully established in the bASCs isolated from two individual bovine donors.

### 3.3 Evaluation of the Characteristics of Gene‐Transduced bASC‐Derived Cell Lines

**Figure 3:**
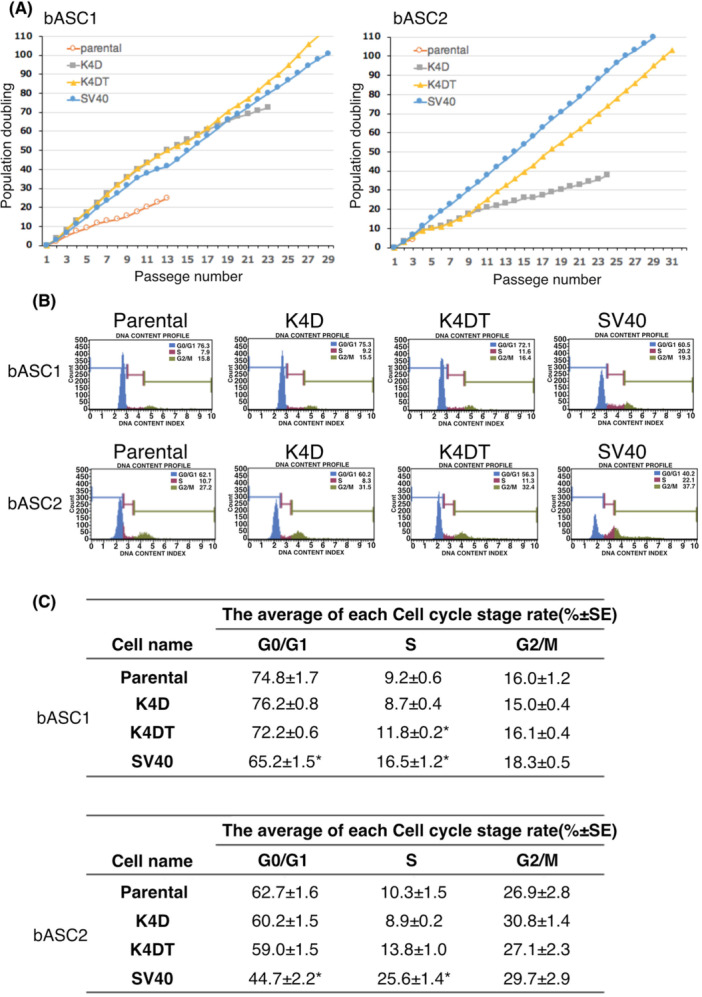
Evaluation of characteristic features of genetically introduced bovine‐derived cells (bASC1 and bASC2). (A) Population doubling levels of parental and cells with the introduced genes. The graph shows accumulated population doubling of parental, K4D, K4DT, and SV40 cells through sequential passaging. (B) A representative histogram of cell cycle analysis of parental, K4D, K4DT, and SV40 cells. (C) Evaluation of the ratio of G0/G1, S phase, and G2/M phases in six samples for each cell group. Statistically significant differences were analyzed using Tukey's multiple comparison test; the *p*‐value is indicated by asterisks in figure (*p < 0.05).

To evaluate proliferative capacity of the generated cell lines, sequential passaging experiments were performed. The parental cells derived from bASC1 and bASC2 had almost stopped proliferating further after reaching cumulative population doublings (PDs) of 24.7 at passage 13 (p13) and 4.2 at passage 2 (p2), respectively. In contrast, K4DT and SV40 cells continued to proliferate until they reached a cumulative PD100, whereas K4D cells ceased proliferating at passages 24 and 25 and did not reach PD100 (Figure 3A). The details of triplicated cell growth of bASC1 are involved as Table S1. This data are available through Figshare (DOI: 10.6084/m9. figshare.32843024).

To assess whether the introduced genes affected cell cycle regulation, cell cycle analysis was performed using the Muse Cell Analyzer. Because parental cells stopped proliferating, the cells at passage 2 were used for the analysis. For the gene‐transduced cell lines, cells derived from bASC1 at passage 12 and cells derived from bASC2 at passage 14 were used for the respective analyses. Comparison of the cell cycle profiles of parental, K4D, and K4DT cells showed no substantial differences in the G0/G1 and G2/M phases, indicating a similar overall distribution among the three cell types (Figure 3B and Figure S3A, S3B). In contrast, SV40 cells exhibited a distinct cell cycle profile, and statistical analysis revealed significant differences in the G0/G1 and S phases compared to the parental cells. No significant differences were observed in the G0/G1 or G2/M phases between the parental cells and either K4D or K4DT cells (Figure 3C).

### 3.4 Karyotype Analysis of K4DT Cells

**Figure 4:**
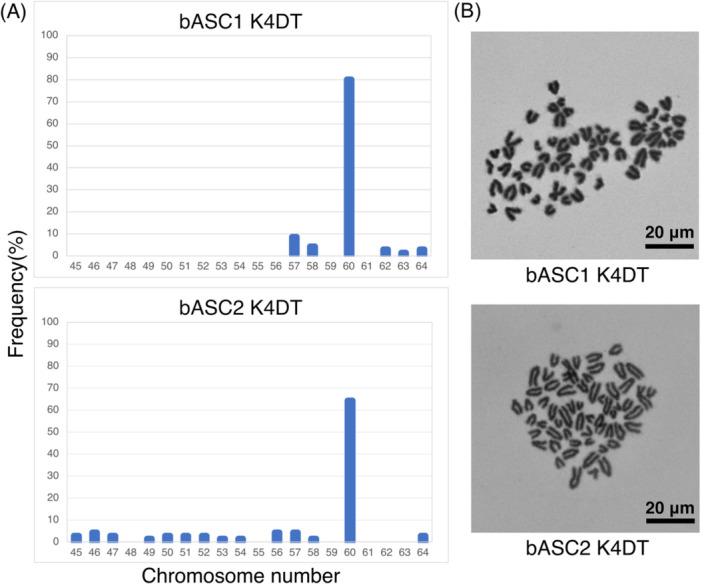
Karyotype analysis of bovine‐derived K4DT cells. (A) Histogram of chromosome number distribution in bASC1_K4DT (P11) and bASC2_K4DT (P13) cells. The x‐axis represents the chromosome number, and the y‐axis indicates frequency of counted metaphase cells. (B) Representative image of chromosome patterns. Scale bar = 20 μm.

For karyotype analysis, chromosome numbers were examined in bASC1_K4DT cells at passage 11 and in bASC2_K4DT cells at passage 13. Chromosomal analysis revealed that K4DT cells derived from both bASC1 and bASC2 contained a mixture of normal (2n = 60) and aneuploid metaphases. In bASC1_K4DT cells, 56 of 70 metaphases (80%) exhibited normal karyotypes, and 46 of 70 metaphases (65.7%) were normal in bASC2_K4DT cells. Both the cell populations exhibited an apparent peak at approximately 60 chromosomes, indicating that the established K4DT cells retained their normal karyotypic structure. Compared with bASC1_K4DT cells, bASC2_K4DT cells displayed a broader range of chromosome number variations (Figure 4A). Despite this variability, both the populations showed an evident peak at 60 chromosomes, indicating that a majority of the cells possessed a near‐normal karyotype (Figure 4B).

### 3.5 Induction of Adipogenic Differentiation in bASCs

**Figure 5:**
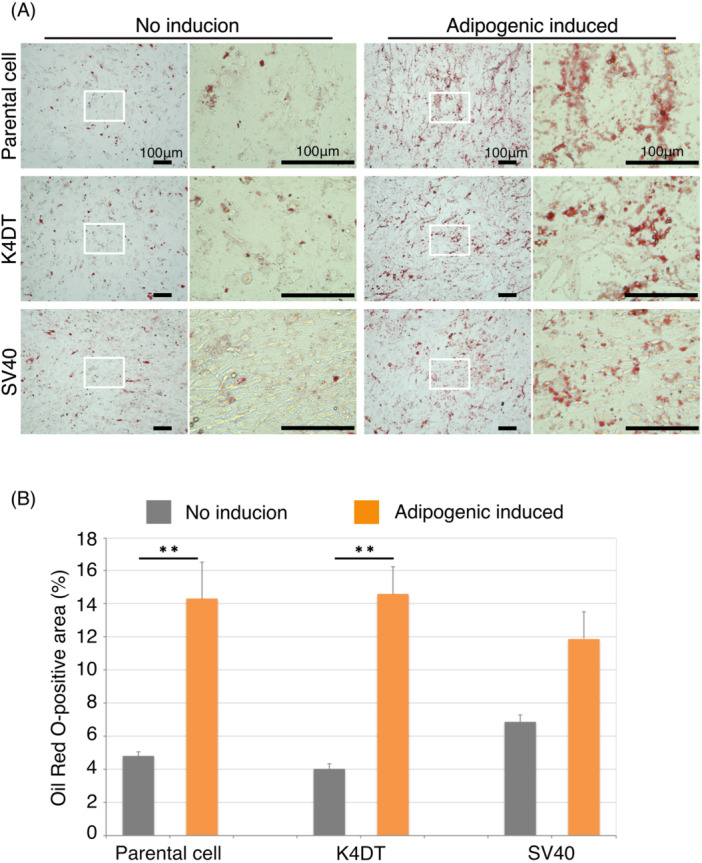
Adipogenic differentiation of parental, K4DT cells and SV40 derived from bASC2 during adipogenic differentiation. (A) Representative Oil Red O staining images of parental, K4DT and SV40 cells cultured in either control medium (No induction) or adipogenic induction medium for 8 days. Low‐magnification (left) and high‐magnification (right) images are shown for each condition. Scale bars = 100 μM. (B) Quantitative analysis of the Oil Red O‐positive area was performed using ImageJ software. Statistical analysis was performed using one‐way analysis of variance (ANOVA) followed by Tukey's multiple comparison test; the P‐value is indicated by asterisks in figure (*p < 0.05; **p < 0.01). Data are presented as the mean ± SE of three independent experiments.

To evaluate whether the established bASC‐derived K4DT cells retained adipogenic differentiation potential, adipogenic induction was performed and intracellular lipid droplet formation was evaluated. Lipid droplets consist of intracellular neutral lipids and can be visualized as red‐stained structures using Oil Red O staining. Oil Red O staining was performed on day 8 after induction of adipogenic differentiation using six cell types: parental cells (bASC1 and bASC2), their corresponding K4DT cells and their corresponding SV40 cells. Representative images of Oil Red O staining are shown in Figure 5A and Figure S4A. In both parental and K4DT cells, adipogenic induction markedly increased the accumulation of Oil Red O‐positive lipid droplets compared with the non‐induced controls. In contrast, SV40 cells also exhibited adipogenic differentiation following induction, although the extent of lipid accumulation appeared to be lower than that observed in the parental and K4DT cells.

Quantitative image analysis of the Oil Red O‐positive area is shown in Figure 5B and Figure S4B. In bASC2, we detected the increased area after the adipogenic induction of parental and K4DT cells (Figure 5B). However, in case of SV40 cells, there was no significance. In bASC1, we observed the increased area after the induction of differentiation. However, we could not obtain the statistical difference in case of bASC1 (Figure S4). bASC1_SV40 cells did not show difference before and after the adipogenic induction (Figure S4B).

## 4 Discussion

In the present study, we established immortalized cell lines from preadipocytes derived from Japanese Black cattle through ectopic expression of CDK4^R24C^, Cyclin D1, and TERT. Furthermore, the established K4DT cells were confirmed to retain their ability to differentiate into adipocytes. Although our research group has previously established immortalized cell lines from several bovine and other animal cell types using the K4DT method, this is the first study in which the K4DT method has been applied to adipose‐derived stromal cells in any species. Compared with parental cells, immortalized cell lines offer several advantages, including a stable proliferative capacity, improved experimental reproducibility, and the ability to perform long‐term functional studies and repeated genetic manipulations. Consequently, the immortalized ASC lines established in this study may provide a stable and reproducible experimental model for investigating adipogenic differentiation and lipid metabolism.

To date, studies on adipocyte immortalization have predominantly relied on viral oncogenes such as the SV40T antigen or human papillomavirus (HPV) E6 and E7. Although these approaches markedly enhance proliferative capacity, they concomitantly inhibit the p53 and pRB pathways, which have been reported to induce genomic instability (Sheppard et al. 1999). Moreover, adipocyte precursor cells immortalized by SV40T have been shown to exhibit limited differentiation capacity (Church et al. 2015).

We have previously succeeded in establishing immortalized cell lines from various species by introducing CDK4^R24C^, Cyclin D1, and TERT, a strategy referred to as the K4DT method (Donai et al. 2014; Gouko et al. 2018). In these studies, using the K4DT method, human dermal papilla cells immortalized were reported to exhibit a loss of endogenous androgen receptor expression (Fukuda et al. 2020). These findings raised concerns that acquisition of a high proliferative capacity might be accompanied by alterations in phenotypes associated with differentiation or functional maintenance. In this context, the observation that bovine adipose‐derived K4DT cells retain an adipogenic differentiation capacity comparable to that of parental cells represents an intriguing outcome of the present study. Furthermore, comparison with SV40 showed that adipogenic differentiation was modestly reduced in the SV40 cells, whereas the K4DT cells maintained differentiation efficiency comparable to that of the parental cells. These findings suggest that the K4DT method may better preserve the adipogenic properties of bovine ASCs than the conventional SV40 method, although further molecular characterization will be required to clarify the underlying mechanisms.

The two ASC‐derived K4DT cell lines independently established from two different cattle in this study did not show substantial differences from parental cells in either the cell cycle or chromosomal analysis. These results suggest that K4DT cells retain functional characteristics comparable to those of the parental cells while maintaining a high proliferative capacity.

Japanese Black cattle have been reported to exhibit a higher intramuscular fat content and greater degree of marbling than Holstein cattle, which are a typical dairy breed. Intramuscular fat is rich in monounsaturated fatty acids (MUFAs), such as oleic acid, which are thought to contribute to improvement of meat texture and flavor as well as development of a Wagyu‐specific aroma (Gotoh et al. 2014). In mature adipocytes, fatty acid composition is regulated in part by stearoyl‐CoA desaturase (SCD), an enzyme that converts saturated fatty acids into MUFAs, and both the expression and enzymatic activity of SCD have been shown to influence the MUFA content (Matsuhashi et al. 2011; Ohsaki et al. 2009). In addition, during adipocyte differentiation, the expression levels of adipogenic transcription factors, including C/EBPβ and C/EBPα, have been reported to be higher in Japanese Black cattle than in Holstein cattle, suggesting that these factors may contribute to breed‐specific differences in intramuscular fat accumulation capacity. Furthermore, PPARγ, which is known as a master regulator of adipocyte differentiation, functions cooperatively with the C/EBP family to play a critical role in adipocyte differentiation and maturation (Ambele et al. 2020). In the present study, the observation that K4DT‐immortalized adipose progenitor cells derived from Japanese Black cattle retained their ability to differentiate into adipocytes suggests that adipogenic transcriptional programs involving PPARγ may be preserved during the differentiation process. In the future, the use of Japanese Black cattle–derived K4DT cells established in this study will be valuable for analyzing the expression of lipid metabolism–related genes, including SCD. Furthermore, applying the K4DT method to Holstein‐derived adipose progenitor cells under identical conditions may provide an experimental platform for comparative analyses of adipogenic characteristics, lipid metabolism, and aroma‐related metabolic pathways between breeds.

Taken together, these results showed that adipose‐derived stromal cells from Japanese Black cattle can be successfully immortalized using the K4DT method with the intact adipogenic differentiation potential. Although further molecular characterization is warranted, the established K4DT cell lines may serve as a stable and reproducible in vitro model for studies of adipogenic differentiation and lipid metabolism in Japanese Black cattle. Although the established in vitro model may provide a useful platform for investigating adipogenesis and lipid metabolism, the information obtained from the cell line is insufficient for the full understanding of the complex process of marbling formation in vivo, which is influenced by multiple genetic factors.

The present study has several limitations that should be acknowledged. The immortalized cell lines were established as population‐derived cultures rather than single‐cell‐derived clones in order to preserve the overall characteristics of the original ASC population, as ASCs are heterogeneous in their proliferative and differentiation capacities. Although this population‐based approach provides a practical model for the initial characterization of immortalized bovine ASCs, future studies should include analyses of individual clones to evaluate clonal variability and confirm genomic stability. To obtain more convincing evidence of the adipogenesis of the immortalized ASC, the establishment of at least five clonal cell lines and biological characterization of each clone such as Western blotting and real‐time PCR of PPARγ and C/EBPα for each individual clone will be required. However, in this study, we could not complete these molecular analyses. The clonal analysis may reveal phenotypic variation among individual clones, such as chromosomal abnormalities. To avoid potential selection artifacts, a larger number of clones might be required to draw definitive conclusions.

## Author Contributions

Rei Sasaki, Hanami Matsukura, Ryo Meguro, and Tomokazu Fukuda performed the experiments and contributed to data analysis. Lanlan Bai, Tomokazu Fukuda, Hiroshi Tomita, and Eriko Sugano contributed to the study design, and mentored and provided technical support for the students. Ken Sawai and Tohru Kiyono provided essential research material for this study. Rei Sasaki and Tomokazu Fukuda wrote the manuscript. All authors read and approved the final manuscript.

## Ethics Statement

All cell cultures used in this study were maintained under standardized laboratory conditions.

## Conflicts of Interest

The authors declare no conflicts of interest.

## Supporting information


**Figure S1:** No crop agarose gel images of the PCR analysis shown in Figure 2A. (A) bASC1, (B) bASC2.

**Figure S2:** No crop western blot images of protein expression shown in Figure 2B. (A) bASC1, (B) bASC2.

**Figure S3:** Cell cycle analysis of immortalized bovine‐derived cells performed in tripricate. (A) bASC1, (B) bASC2. (C) Representative unprocessed Muse Cell Analyzer dot plots corresponding to the cell cycle histograms shown in Figure 3B.

**Figure S4:** Adipogenic differentiation of parental, K4DT cells and SV40 derived from bASC1 during adipogenic differentiation.

**Table S1:** Raw cell‐counting data used to calculate population doubling (PD) values for each experimental group (Parental, K4D, K4DT and SV40).

## Data Availability

The data that support the findings of this study are available from the corresponding author upon reasonable request.
