# Supplementary material for: Immortalization of Adipose‐Derived Stromal Cells From Japanese Black Cattle
Source: Cell Biol Int. 2026 Aug 6;50(8):e70194. doi: 10.1002/cbin.70194 (PMC13445999; doi:10.1002/cbin.70194)
Supplement: Supplementary file 1 [file CBIN-50-0-s001.docx]

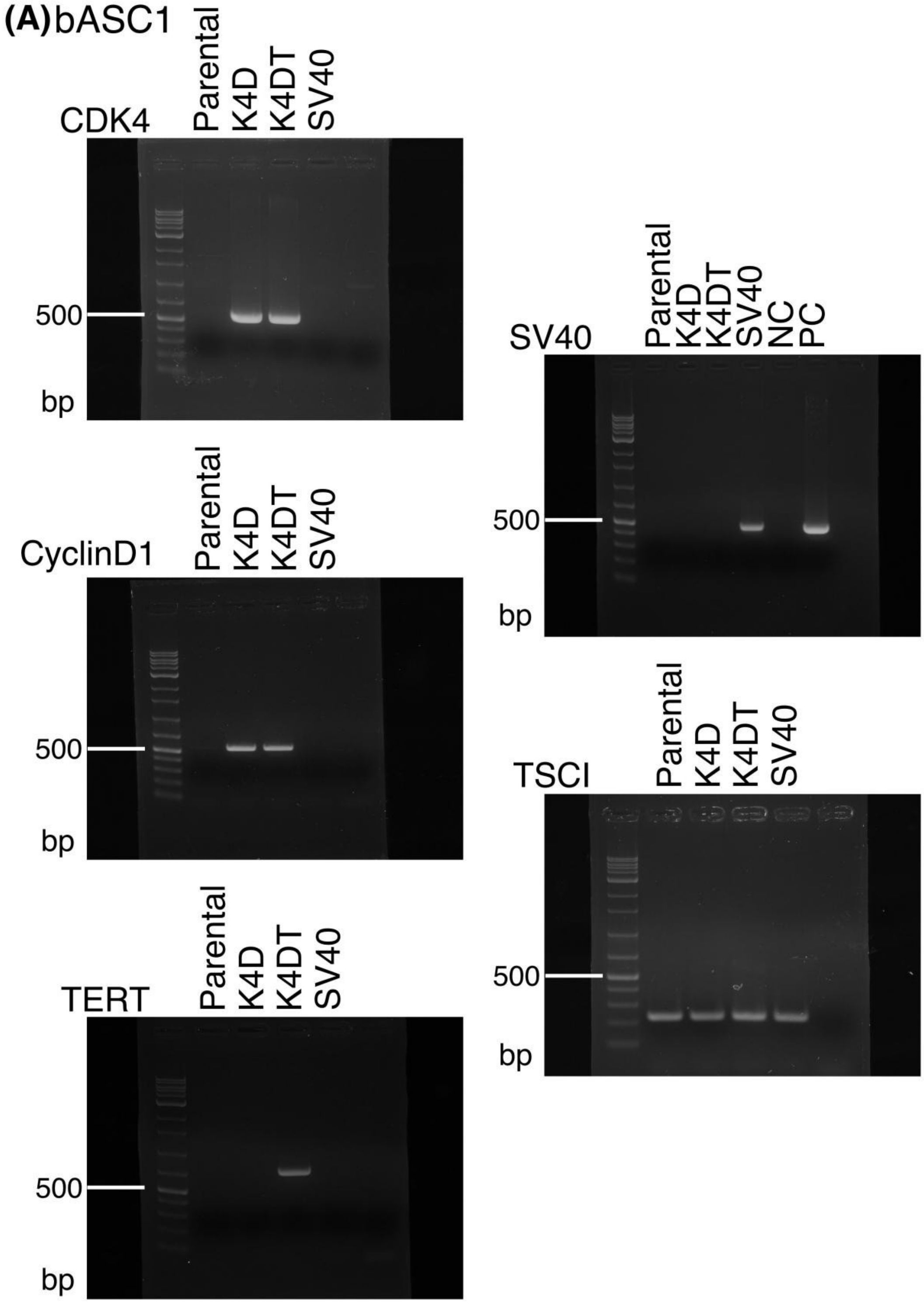

(A) bASC1
CDK4
Parental
K4D
K4DT
SV40
500
bp
CyclinD1
Parental
K4D
K4DT
SV40
500
bp
TERT
Parental
K4D
K4DT
SV40
500
bp
SV40
Parental
K4D
K4DT
SV40
NC
PC
500
bp
TSCI
Parental
K4D
K4DT
SV40
500
bp

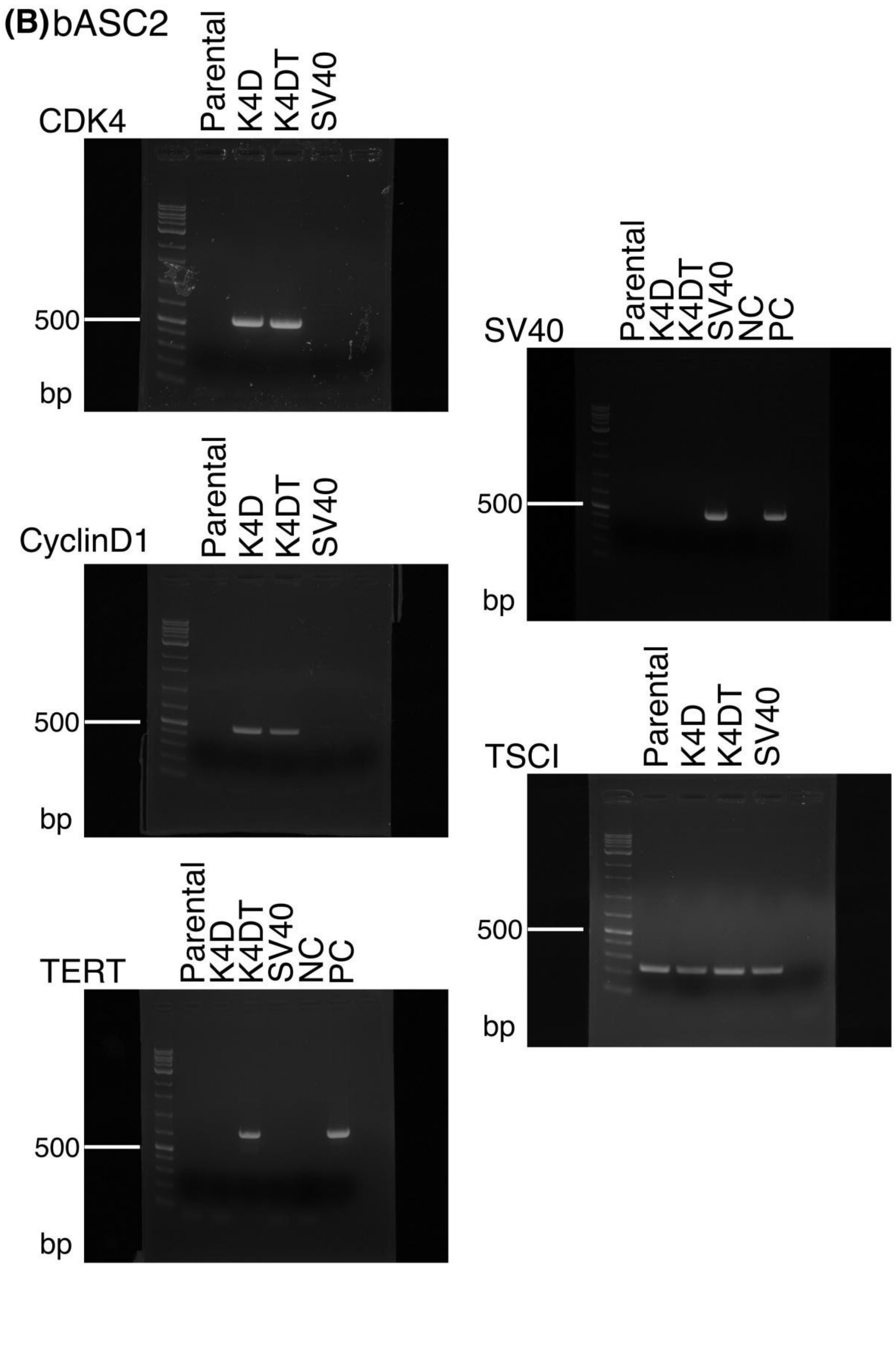

(B) bASC2
CDK4
Parental
K4D
K4DT
SV40
500
bp
CyclinD1
Parental
K4D
K4DT
SV40
500
bp
TERT
Parental
K4D
K4DT
SV40
NC
PC
500
bp
SV40
Parental
K4D
K4DT
SV40
NC
PC
500
bp
TSCI
Parental
K4D
K4DT
SV40
500
bp
