# Supplementary material for: Immortalization of Adipose‐Derived Stromal Cells From Japanese Black Cattle
Source: Cell Biol Int. 2026 Aug 6;50(8):e70194. doi: 10.1002/cbin.70194 (PMC13445999; doi:10.1002/cbin.70194)
Supplement: Supplementary file 2 [file CBIN-50-0-s004.docx]

(A) bASC1

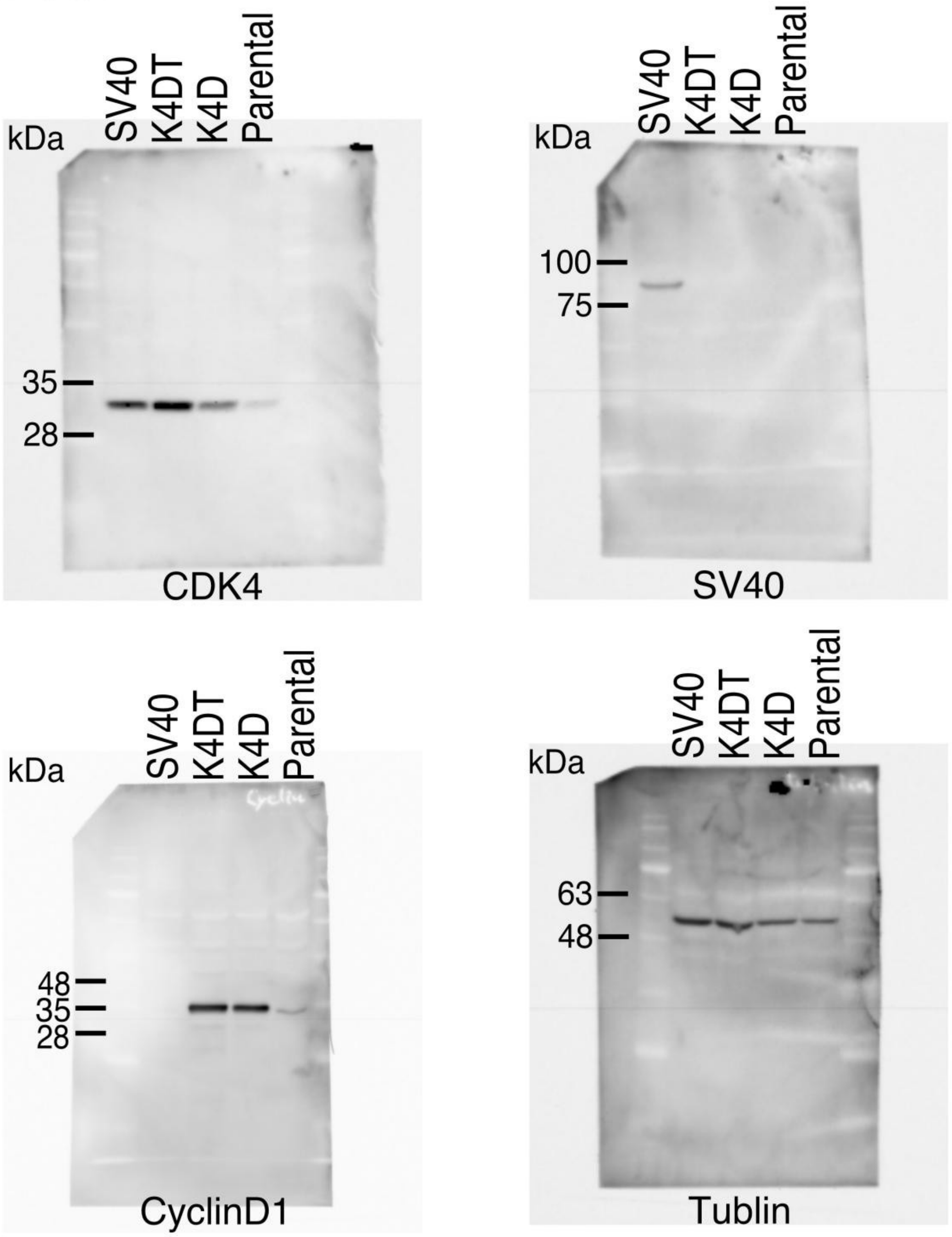

kDa
SV40
K4DT
K4D
Parental
35
28
CDK4
kDa
SV40
K4DT
K4D
Parental
100
75
SV40
kDa
SV40
K4DT
K4D
Parental
48
35
28
CyclinD1
kDa
SV40
K4DT
K4D
Parental
63
48
Tublin

(B) bASC2

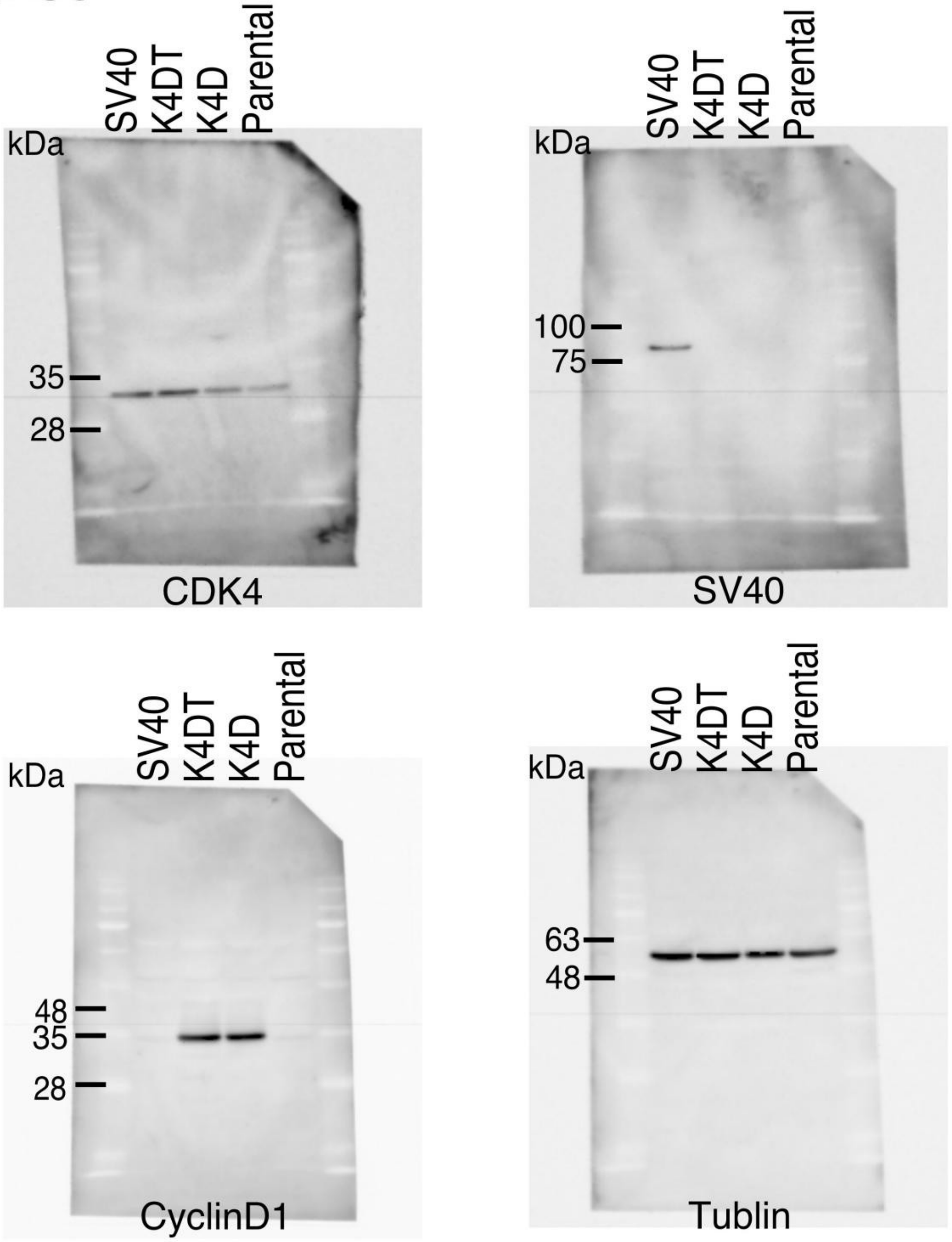
SV40
K4DT
K4D
Parental
kDa
35
28
CDK4
SV40
K4DT
K4D
Parental
kDa
100
75
SV40
SV40
K4DT
K4D
Parental
kDa
48
35
28
CyclinD1
SV40
K4DT
K4D
Parental
kDa
63
48
Tublin
