# Supplementary material for: Immortalization of Adipose‐Derived Stromal Cells From Japanese Black Cattle
Source: Cell Biol Int. 2026 Aug 6;50(8):e70194. doi: 10.1002/cbin.70194 (PMC13445999; doi:10.1002/cbin.70194)
Supplement: Supplementary file 3 [file CBIN-50-0-s005.docx]

(A) bASC1

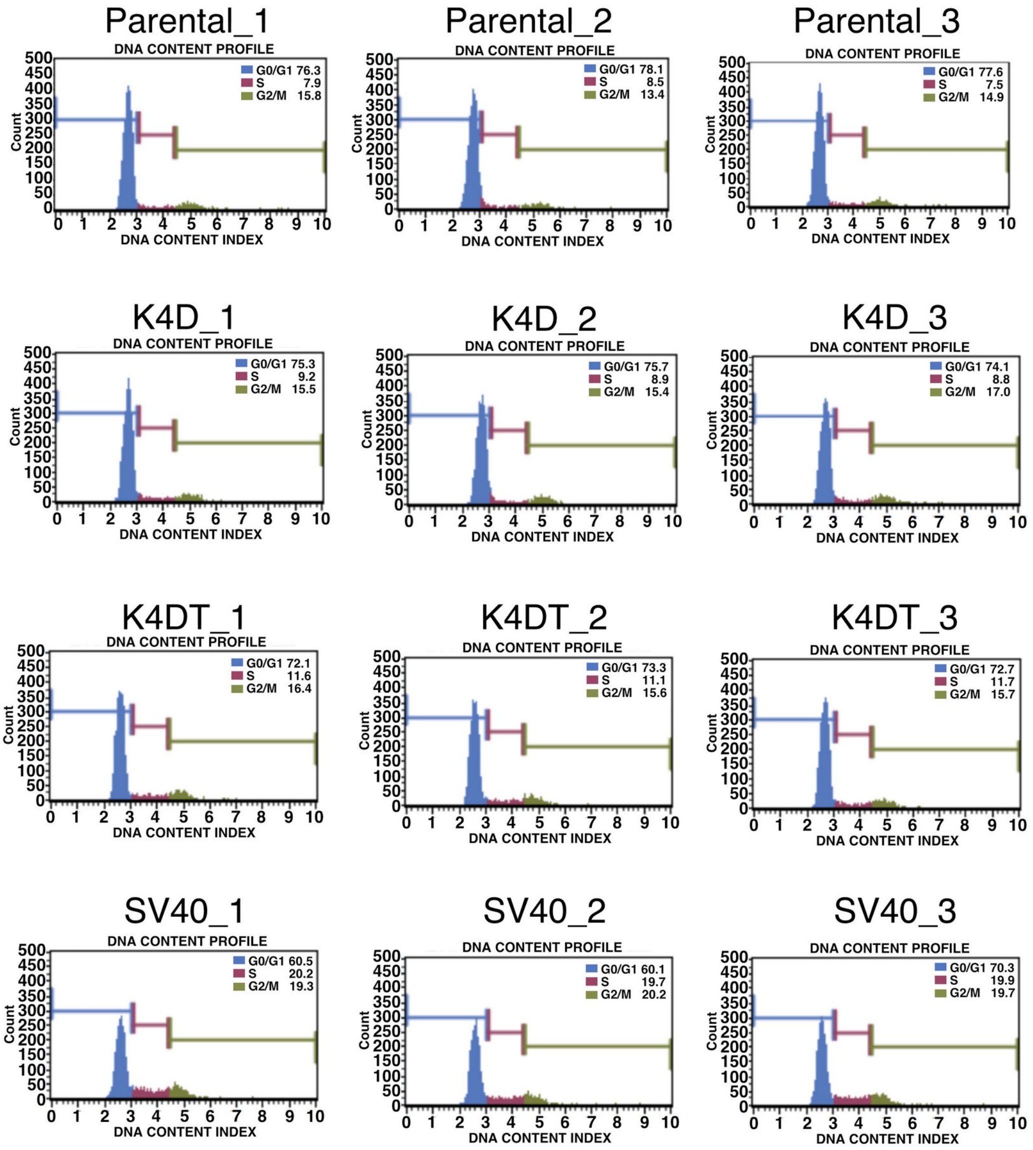

Parental_1
DNA CONTENT PROFILE
G0/G1 76.3
S 7.9
G2/M 15.8
Count
DNA CONTENT INDEX
Parental_2
DNA CONTENT PROFILE
G0/G1 78.1
S 8.5
G2/M 13.4
Parental_3
DNA CONTENT PROFILE
G0/G1 77.6
S 7.5
G2/M 14.9
K4D_1
DNA CONTENT PROFILE
G0/G1 75.3
S 9.2
G2/M 15.5
K4D_2
DNA CONTENT PROFILE
G0/G1 75.7
S 8.9
G2/M 15.4
K4D_3
DNA CONTENT PROFILE
G0/G1 74.1
S 8.8
G2/M 17.0
K4DT_1
DNA CONTENT PROFILE
G0/G1 72.1
S 11.6
G2/M 16.4
K4DT_2
DNA CONTENT PROFILE
G0/G1 73.3
S 11.1
G2/M 15.6
K4DT_3
DNA CONTENT PROFILE
G0/G1 72.7
S 11.7
G2/M 15.7
SV40_1
DNA CONTENT PROFILE
G0/G1 60.5
S 20.2
G2/M 19.3
SV40_2
DNA CONTENT PROFILE
G0/G1 60.1
S 19.7
G2/M 20.2
SV40_3
DNA CONTENT PROFILE
G0/G1 70.3
S 19.9
G2/M 19.7

(B) bASC2

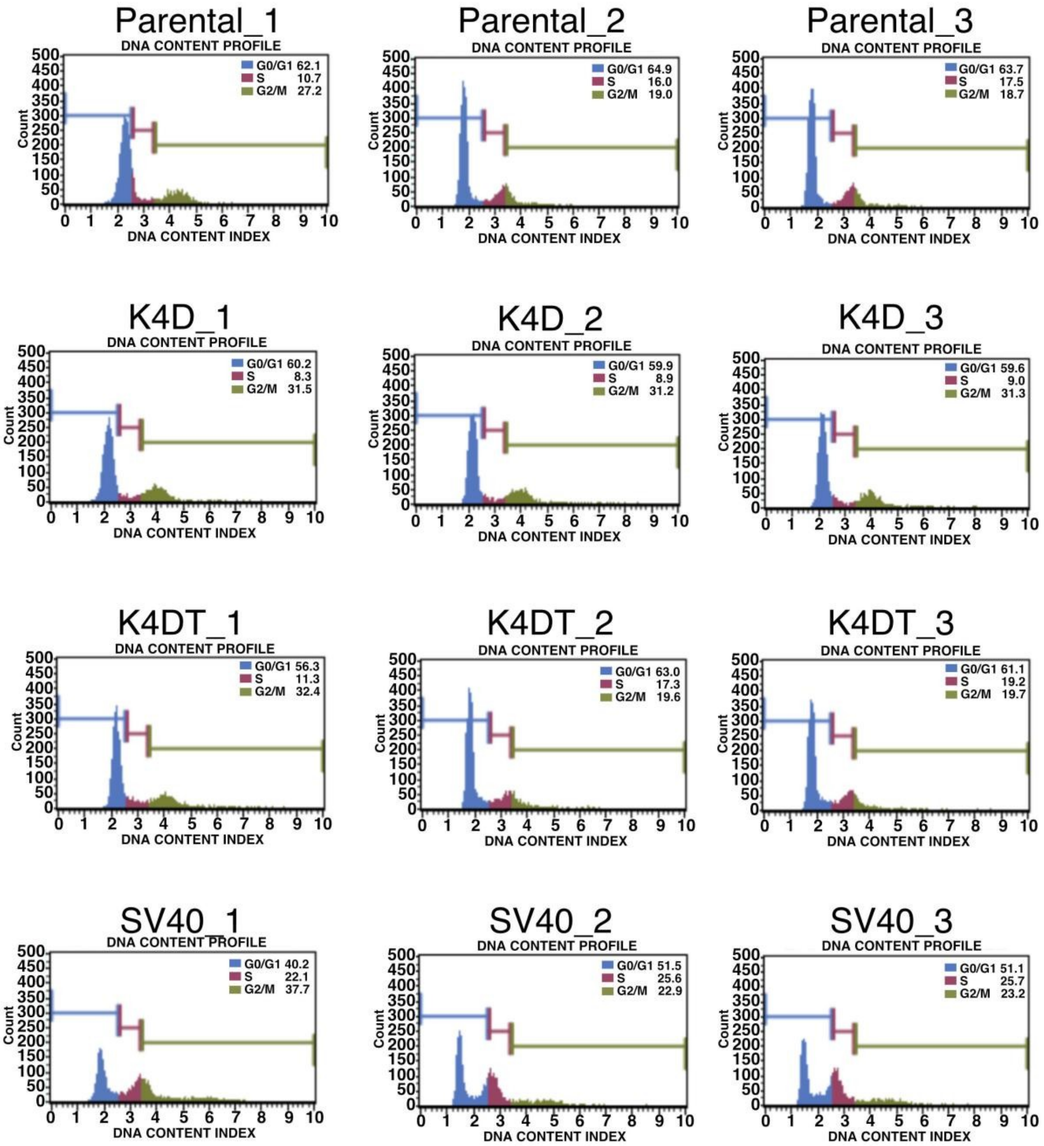

Parental_1
DNA CONTENT PROFILE
G0/G1 62.1
S 10.7
G2/M 27.2
Parental_2
DNA CONTENT PROFILE
G0/G1 64.9
S 16.0
G2/M 19.0
Parental_3
DNA CONTENT PROFILE
G0/G1 63.7
S 17.5
G2/M 18.7
K4D_1
DNA CONTENT PROFILE
G0/G1 60.2
S 8.3
G2/M 31.5
K4D_2
DNA CONTENT PROFILE
G0/G1 59.9
S 8.9
G2/M 31.2
K4D_3
DNA CONTENT PROFILE
G0/G1 59.6
S 9.0
G2/M 31.3
K4DT_1
DNA CONTENT PROFILE
G0/G1 56.3
S 11.3
G2/M 32.4
K4DT_2
DNA CONTENT PROFILE
G0/G1 63.0
S 17.3
G2/M 19.6
K4DT_3
DNA CONTENT PROFILE
G0/G1 61.1
S 19.2
G2/M 19.7
SV40_1
DNA CONTENT PROFILE
G0/G1 40.2
S 22.1
G2/M 37.7
SV40_2
DNA CONTENT PROFILE
G0/G1 51.5
S 25.6
G2/M 22.9
SV40_3
DNA CONTENT PROFILE
G0/G1 51.1
S 25.7
G2/M 23.2
Count
DNA CONTENT INDEX

(C)

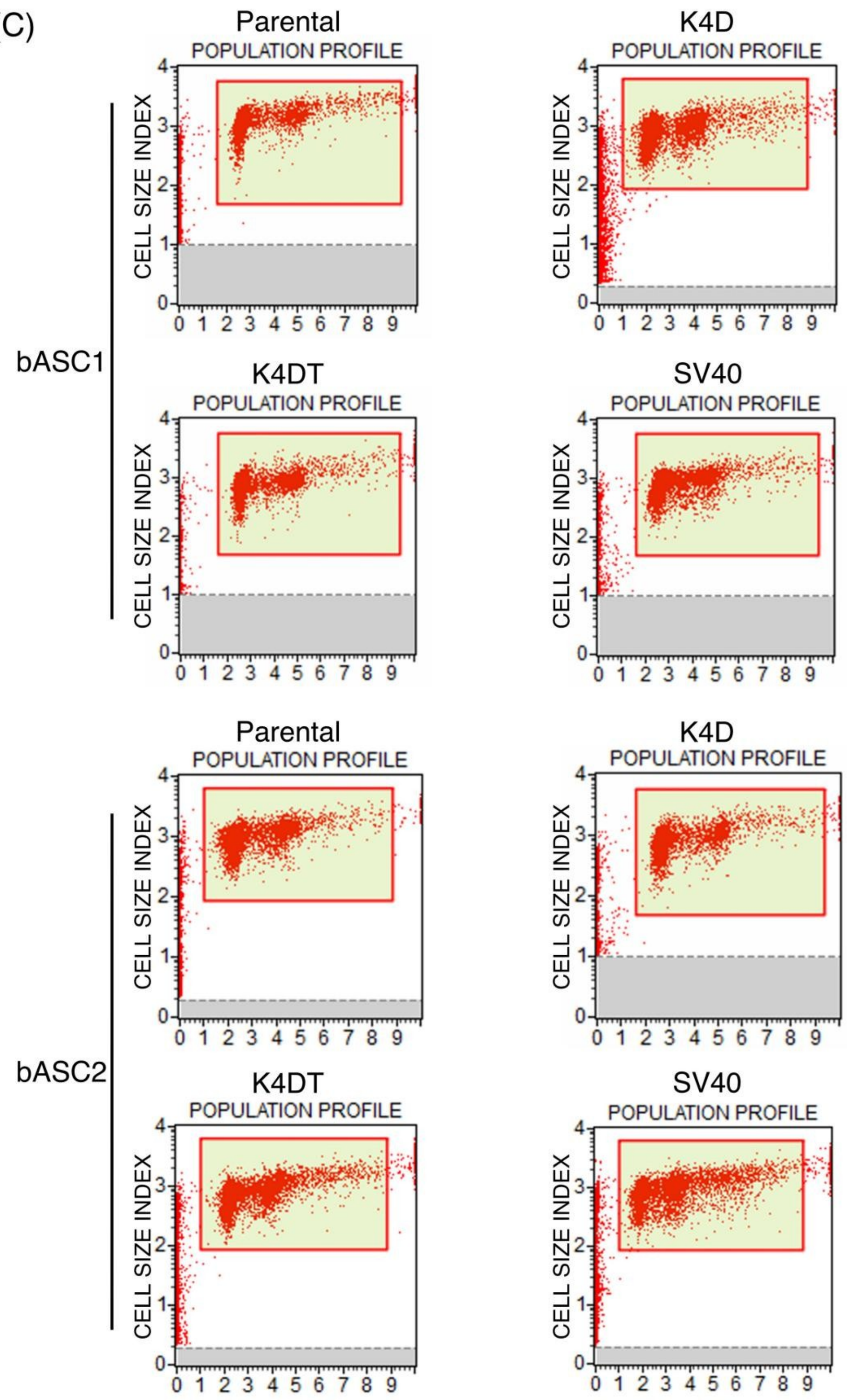
bASC1
Parental
POPULATION PROFILE
CELL SIZE INDEX
K4D
POPULATION PROFILE
CELL SIZE INDEX
K4DT
POPULATION PROFILE
CELL SIZE INDEX
SV40
POPULATION PROFILE
CELL SIZE INDEX
bASC2
Parental
POPULATION PROFILE
CELL SIZE INDEX
K4D
POPULATION PROFILE
CELL SIZE INDEX
K4DT
POPULATION PROFILE
CELL SIZE INDEX
SV40
POPULATION PROFILE
CELL SIZE INDEX
